# Effect of clinical pharmacist encounters in the transitional care clinic on 30-day re-admissions: A retrospective study

**DOI:** 10.3934/publichealth.2019.3.345

**Published:** 2019-09-24

**Authors:** Panid Borhanjoo, Priscile Kouamo, Mafuzur Rahman, Margaret Norton, Madhavi Gavini

**Affiliations:** 1 Department of Internal Medicine, State University of New York, Downstate Medical Center, Brooklyn, N.Y. 11213, U.S.A.; 2 School of Public Health, State University of New York, Downstate Medical Center, Brooklyn, N.Y., U.S.A.; 3 Department of Family Medicine, State University of New York, Downstate Medical Center, Brooklyn, N.Y., U.S.A.

**Keywords:** transitional-care clinic (TCC), affordable care act (ACC), clinical pharmacist (CP), 30-day re-admission, electronic medical record (EMR)

## Abstract

Hospitalized patients who meet specific criteria at discharge are referred to the transitional care clinic team consisting of a nurse practitioner and/or physician and a clinical pharmacist. In collaboration with the providers, the pharmacist reviews medications for appropriateness, assesses adherence, recommends medication changes and provides education. The purpose of this study was to measure the effect of an outpatient transitional care clinical pharmacist on 30-day re-admissions in an urban setting serving a population of low socioeconomic status. After receiving IRB approval, this single-center retrospective study analyzed records of 573 patient visits of which nearly 75% included a clinical pharmacist interaction. Rates of 30-day re-admissions were not statistically different among the two groups, however, it was found that each added co-morbidity significantly increased the patients' 30-day re-admission rate by 26%.

## 1. Introduction

Thirty-day hospital re-admissions cost Medicare an additional U.S $17.5 billion annually [1]. For that reason, the United States government created new policies to penalize hospitals with higher re-admission rates [2] and financially incentivize healthcare administrators to improve patients' health and reduce hospital re-admission. Many hospital re-admissions are prevented by improving patients' quality of care during, before and after discharge [3]. Numerous studies have been published on the effect of pharmacist interventions in the transitions of care through medication reconciliation, counseling and follow-up phone calls. These studies revealed a trending decrease in the percentage of patients re-admitted within 30 days post hospital discharge but additional studies were needed to reach a stable conclusion [3]–[6] Arnold et al. showed that there is a reduced re-admission rate in patients followed by a pharmacist post discharge compared to patients who receive only standard of care (9.2% vs. 19.4%) [5]. Another study showed a significant reduction in 180-day total healthcare cost in the intervention group with a pharmacy based transitions of care program compared to the usual post-discharge care [7]. Pharmacist involvement through counseling and medication reconciliation has demonstrated the potential to reduce the re-admission rates but was not found to be statistically significant [8]. However, there was a statistically significant reduction in re-admission rates between patients who had a pharmacist face to face visit versus those who only had a follow-up phone call with the pharmacist. Other studies also showed a decreased 30-day re-admission in high-risk patients with pneumonia, heart failure or acute myocardial infarction [9]–[12]. A systematic review and meta-analysis showed that pharmacist interventions during transitions of care reduce re-admission rates by 19% compare to the usual care [13].

In our institution, the University Hospital of Brooklyn (UHB), the Transitions of Care Clinic (TCC) was implemented as a pilot project to address UHB's high hospital re-admission rates. Our institution located in central Brooklyn serves patients from one of the nation's poorest and most challenging health care environments with respect to health status and access. This dense, urban area is populated by a low-income, predominantly Caribbean-American population. Many of our patients lack appropriate social, financial, clinical support systems to reduce hospital re-admissions. Over the last few years, CMS began to levy penalty for 30-day or 15-day re-admissions. In addition to this penalty, Medicare may request refunds for re-admissions previously paid for. The Transition of Care Clinic (TCC) was one of such measures implemented to improve patient care and address issues most implicated for re-admissions. Hospitalized patients who met a set of specific criteria with high likelihood of re-admission were referred to the TCC at discharge and managed with additional resources to address specific diagnoses including congestive heart failure (CHF), acute myocardial infarction (AMI), pneumonia, and diabetes mellitus (DM). In the TCC, a provider (physician or nurse practitioner), and a clinical pharmacist collaborated to manage patients for two half-day sessions weekly. The team ensured that patients were adherent to the discharge plan and had appropriate follow-up care. The clinical pharmacist reconciled medications, reviewed medications for appropriateness, assessed adherence to medications and provided education in collaboration with the provider. Through this study, the effectiveness of an outpatient transitional care CP was measured by comparing 30-day re-admission rates for patients who had a CP encounter during their TCC visit versus those who did not. The effects of number of interventions and patients' comorbidities on the 30-day re-admission rates were also evaluated.

## 2. Methods

The study received expedited review from the SUNY Downstate Medical Center Institutional Review Board Committee in April 2018. The study was a single center retrospective and included all adult patients seen at the SUNY Downstate TCC from January 1, 2016 to December 31, 2017. A retrospective chart review was performed on patients who met the study's inclusion criteria, to determine what percentage of patients seen in the TCC by the provider and the CP are re-admitted to our hospital within 30 days compared to patients who were seen only by the provider during the clinic visit. Patients were identified through a computer-generated list from the electronic medical record (EMR). Patients who were seen by the provider and the CP were the intervention group. The control group consisted of patients who were not seen by a pharmacist either due to CP's absence or because provider decided CP encounter was unnecessary. The number and the type of CP interventions were determined using the report generated from the CP intervention database Quantifi^®^. For CP encounters with no corresponding entry in the Quantifi^®^ report, interventions were identified from the CP's patient encounter notes in the electronic medical record. Number of patient co-morbidities were obtained from the EMR. The 30-day re-admission was determined from a report generated by the institution's information technology department. Patient data collected included age, gender, number of comorbidities, insurance code, date of admission, reason for admission, date of discharge, number of days admitted, 30-day re-admission, date of re-admission, reason for re-admission, number of pharmacist interventions and common pharmacist interventions (medication reconciliation, patient education/counseling, identified medication non adherence, discontinue therapy, initiating therapy).

A total of 573 patient encounters in the TCC were reviewed, with 422 including a CP encounter and 151 without (Table 1). The primary endpoint was composite difference in 30-day re-admission rates between patients who had a CP encounter and those who did not. Secondary endpoints of interest were the effect of total number of CP interventions at each visit, top five pharmacist interventions and number of patient co-morbidities on the 30-day re-admission rate.

### 2.1. Data analysis

Data collected was entered in Microsoft^®^ Excel. Quantitative analysis procedures were utilized to analyze the data collected from the survey using SAS 9.4. Fisher exact test was used to test the association of variables in a two-way frequency table. The trend test was done using Mantel-Haenszel test.

Logistic regression was used to predict 30-day re-admission (for any reason) from encounter with the CP, patient age, gender and number of comorbidities. Tests of utility of polynomial terms in scored predictors and of predictor interactions were conducted; the Hosmer-Lemeshow lack of fit test was applied.

**Table 1.** Subject population demographics and characteristics.

(N = 573)	Patients seen by pharmacist N = 422 (%)	Patients not seen by pharmacist N = 151 (%)	*p*-value
Age range in years	19–97	23–94	**0.02**
Gender			1.00
(Female)	230 (54.5)	82 (54.3)	
(Male)	192 (45.5)	69 (45.7)	
Insurance			0.09
Medicare	226 (53.5)	93 (61.6)	
Medicaid	72 (17.1)	14 (9.3)	
Self-pay	25 (5.9)	8 (5.3)	
Other	99 (23.5)	36 (23.8)	
No. of comorbidities			0.06
0–3	235 (55.7)	101 (66.8)	
4–7	174 (41.2)	46 (30.5)	
8–10	13 (3.1)	4 (2.7)	
Reason for admission			
Congestive heart failure	66 (15.64)	12 (7.95)	**0.02**
Diabetes mellitus	36 (8.53)	11 (7.28)	0.57
Hypertension	16 (3.79)	6 (3.97)	0.98
Coronary artery disease	34 (8.06)	10 (6.62)	0.51
Chronic obstructive pulmonary disease	14 (3.32)	6 (3.97)	0.81
Asthma	5 (1.18)	2 (1.32)	0.93
Kidney disease	13 (3.08)	8 (5.30)	0.28
Venous thromboembolism	21 (4.98)	2 (1.32)	0.06
Infectious disease	55 (13.03)	26 (17.22)	0.24
Other	162 (38.39)	68 (45.03)	0.15

Data are presented as whole numbers and percentages in parentheses. *p*-values calculated using Wilcoxon 2-sided test (age, comorbidities), Pearson 2-sided chi-square test (gender and insurance) and Mann-Whitney test (reasons for admission). Significant values are bolded.

## 3. Results

Data was obtained and analyzed for 573 records; 422 (73.65%) of patient visits included a CP encounter and 151 (26.35%) did not. Forty-six (8.03%) out of 573 patients seen in the TCC had 30-day re-admissions (Table 2). Of these 46, 25 (4.36%) were re-admitted for the same reason as original admission and 21 (3.66%) were re-admitted for a different reason. Thirty-two (69.56%) of the 46 were seen by the CP and 14 (30.44%) were not (*p* = 0.49). Thirty-day re-admission rates for patients seen by the CP was 7.58% (32/422) and those not seen by the CP was 9.27% (14/151). Of the 7.58% re-admitted patients whom were seen by a CP, 4.50% were re-admitted for the same reason (as original admission diagnosis) and 3.08% were re-admitted for other reasons. Of the 9.27% of patients re-admitted not seen by a pharmacist, 3.97% were re-admitted for the same reason and 5.30% were re-admitted for other reasons. For the secondary outcomes, there was no significant difference in 30-day re-admissions based on the total number of CP interventions (*p* = 0.30) (Table 3). Also, there was no statistically significant difference in 30-day re-admission rates for each of the top five interventions; medication reconciliation, patient education, non-adherence identification, therapy discontinuation and initiation had *p*-values of 0.50, 0.61, 0.72, 0.28 and 0.82 respectively. (Table 4). Using logistic regression, the only significant independent predictor of 30-day re-admission was found to be the number of comorbidities (adjusted odds ratio 1.26, 95% confidence interval 1.07–1.47, *p* = 0.005). There was a 26.00% increased chance of 30-day re-admission for each added co-morbidity and results were similar when re-admissions for same reason, or for a different reason were excluded.

**Table 2.** Clinic encounters and 30-day re-admissions rates.

	30-day re-admissions	No 30-day re-admissions	30-day re-admissions for same reason	30-day re-admissions for other reason
Seen by the pharmacist (%) (N = 422)	32 (7.58)	390 (92.42)	19 (4.50)	13 (3.08)
Not seen by the pharmacist (%) (N = 151)	14 (9.27)	137 (90.73)	6 (3.97)	8 (5.30)
Total (N = 573)	46 (8.03)	527 (91.97)	25 (4.36)	21 (3.66)

Data are presented as whole numbers and percentages in parentheses. *p*-values calculated using Mann-Whitney test and all values were not significant (> 0.05).

**Table 3.** Pharmacist interventions and 30-day re-admission.

Number of pharmacist interventions	30-day re-admissions (%)	No 30-day re-admissions (%)	Total number of patients N = 573 (%)
None/not seen	14 (9.27)	137 (90.73)	151 (26.35)
1–3	21 (9.68)	196 (90.32)	217 (37.87)
4–6	8 (4.97)	153 (95.03)	161 (28.0)
7–9	2 (7.41)	25 (92.59)	27 (4.71)
≥ 10 (10–14)	1 (5.88)	16 (94.12)	17 (2.97)
Total	46 (8.03)	527 (91.97)	573 (100.00)

Data are presented as whole numbers and percentages in parentheses. *p*-values calculated using Mann-Whitney test and all values were not significant (> 0.05).

**Table 4.** Top five pharmacist interventions and 30-day re-admission N = 573 (%).

Pharmacist intervention		30-day re-admission for any reason N = 46 (%)	No 30-day re-admission N = 527 (%)
Medication reconciliation	Yes	30 (65.22)	370 (70.21)
	No	16 (34.78)	157 (29.79)
Patient education	Yes	31 (67.39)	375 (71.16)
	No	15 (32.61)	152 (28.84)
Medication non-adherence	Yes	13 (28.26)	133 (25.24)
	No	33 (71.74)	394 (74.76)
Discontinue therapy	Yes	4 (8.70)	82 (15.60)
	No	42 (91.30)	445 (84.40)
Initiate therapy	Yes	6 (13.04)	66 (12.53)
	No	40 (86.96)	461 (87.48)

Data are presented as whole numbers and percentages in parentheses. Data are presented as whole numbers and percentages in parentheses. *p*-values calculated using Mann-Whitney test and all values were not significant (> 0.05).

## 4. Discussion

There has been a major transformation in public health sector with the implementation of the Affordable Care Act (ACA) and its component Hospital Re-admissions Reduction Program (HRRP) in 2012. The 30-day re-admission penalties imposed by these programs (over 2 billion USD as of 2018) has led to introduction of many health care models aimed to reduce re-admission rates [2]. Transitional care management processes have been tested and proven promising in reducing 30-day re-admission rates particularly if paired with re-admission risk-assessment tools and patient-specific interventions guided by disease-specific measures in a multi-disciplinary care delivery team [2],[14],[15].

Our study did not meet the primary outcome and did not demonstrate a statically significant reduction in 30-day re-admission rate in patients who had an encounter with a CP in the TCC. This was in part due to lack of power. In order to have achieved an 87% power for a 2-sided Fisher exact test at significance level = 0.05, an intervention group of 507 and control group of 168 were needed (compared to 390 and 137 respectively). Simple organizational-level interventions such as timely documentations, follow-up appointments and medication-related interventions alone do not necessarily reduce re-admission rates [16],[17]. This is likely because in order to effectively transition care from inpatient setting to community, complex interventions using multidisciplinary care plans and patient-tailored interventions are necessary [18]. Transitional care CP interventions such as medication reconciliation, allergy clarifications, dosing adjustments and cost-analysis have been proven to reduce morbidity/mortality on top of potential adverse effect reduction [19]. Multidisciplinary care systems managed by inpatient and TCC CPs have been implemented in the past with somewhat promising results [19]. Current literature suggests that the combination of organizational-level interventions such as medication-management, timely admission/discharge documentation, post-discharge telephone follow-up and in-home visits combined with a multi-disciplinary care delivery team consisting of a provider, CPs and social worker were particularly potent means of reduction in re-admission rates [19]–[22]. In contrast, many studies, including one small (n = 62) case-cohort study in the UK and a systematic review have failed to show re-admission rate reductions in patient's receiving a single intervention alone post-discharge [23]–[25]. Pharmacy-based transitional care services can be associated with composite reduction in 30-day and 180-day re-admission rates respectively [26], particularly when interventions transcend beyond medication-related services and include discharge plan reinforcement, appointment assistance (specialist authorizations, transport arrangements) and resolving insurance-related problems [23]. Such higher-dimension care delivery models managed by outpatient CPs prove to reduce re-admissions when tailored to the population they serve, particularly, communities with a low socioeconomic status. The TCC care team including the CP provided patient-tailored care with the majority of the time spent in discharge plan reinforcement and follow-up appointment assistance and coordination. Re-admission was likely due to the complexity of the patient population, social issues and other limitations specific to our patient population.

Another possible explanation for lack of reduction in 30-day re-admissions was challenges pertaining to resource availability and our community-based health/access status. Our TCC clinic is utilized for patients with a wide range of re-admission risks and visits were usually a one-time encounter unless the patients needed an additional follow-up. Often times the clinic is used to schedule early follow-ups for patients with perceived high re-admission risks solely due to medication non-adherence/access or poor social support system. Furthermore, given the multitude of problems among our patient population, TCC CPs were limited by the number of problems they were able to address for each patient in a particular clinic encounter. Our re-admission rates could have perhaps been reduced if patients referred to TCC for 7-day post-discharge appointments were high risk patients (re-admission risk > 20%) as demonstrated in a study in North Carolina using 44,473 unique Medicaid recipients with 65,085 discharged. This study showed true benefit for early follow-up within 7 days for patients with re-admission risk > 20% [27]. One pilot study of 34 patients showed that CP-led transitional care targeted towards high risk patients using LACE+ re-admission scoring system prompted a higher level of effective multidisciplinary care team and reduced 30 and 90-day re-admission rates [25]. This was particularly true when high-risk patients were identified early using re-admission risk assessment scoring systems [26]. The argument can be made that more efficient and high-quality multidisciplinary care can be provided if fewer patients who are high risk are referred to TCC. While many care-delivery models exist and have been proven to reduce re-admission rates if implemented with the right parameters, it is critical to consider the limitations health care entities face based on the populations they serve and lack of resources.

### 4.1. Secondary end point

Beyond composite 30-day reduction rates, the effect of CP interventions at each visit and the number of patient co-morbidities as a predictor of re-admission rates were also examined. There was no significant difference in 30-day re-admissions based on the total number of CP intervention sand each of the top five CP interventions at each encounter. A strong patient-pharmacist relationship with a positive rapport serves as a vital component of an effective reconciliation process and population demographics, their perception of CPs and cultural barriers may serve to hinder this course [24]. Also, as CPs are more involved in the transitional care of complex patients, the importance of above-mentioned relationships becomes more prominent. The only significant independent predictor of 30-day re-admission was the number of comorbidities (adjusted odds ratio 1.26, *p* = 0.005) and re-admission rates increased 26% for each additional comorbidity. The total number of comorbidities appear to be a stronger predictor of the re-admission rates as opposed to the acute illness severity [28]–[30]. This increased number of comorbidities necessitates a care delivery that is both personalized and collaborative in order to effectively reduce 30-day re-admissions [31].

There were some limitations in this study. The power of study was limited as described above. The number of patients who were not seen by the CPs (control group) was 151 compared to the intervention group (N = 422). Moreover, the age difference between the 2 groups was significantly different. This was mainly because the control group was not pre-designated and randomized, and the 2 groups were partially standardized based on patients' characteristics and comorbidities. This discrepancy and mismatch could have underestimated the true 30-day re-admission rate in the control group. Moreover, the intervention group had 174 subjects with 4–7 comorbidities whereas the control group had 46. This difference in patient complexity may have also led to a higher 30-day re-admission rate in the intervention group. Another possible limitation was re-admission rates beyond 30 days post-discharge as one study did demonstrate a reduction at 180 days [23]. Another limitation was that the emergency room visits within 30-days following the discharge were not counted as “30-day re-admissions” though many of such visits were related to the original admission diagnosis. Most of the patients in the community had multiple social issues which was mostly managed by the care time as a social worker was not part of the team. Lastly, many of our frequently re-admitted patients (super utilizers) visited multiple emergency rooms in the borough and records of those re-hospitalizations were not available.

## 5. Conclusion

There was no significant difference in the 30-day re-admissions in patients who had a pharmacist encounter and those who did not. The rate of 30-day re-admissions increased with the number of patient co-morbidities. Future studies are warranted to further examine the effective role of transitional care CPs to reduce hospital re-admission rates in similar urban/low socioeconomic communities.
